# The association between long emergency department stays and inpatient length of stay: a retrospective cohort study

**DOI:** 10.1186/s12873-026-01537-4

**Published:** 2026-03-16

**Authors:** David A. Firth, Muhammad Adeel Baig, Betsy Teresa, Gioacchino Cracolici, Adrian Boyle

**Affiliations:** 1 Department of Emergency Medicine, Cambridge University Hospital, Cambridge, UK; 2 Department of Emergency Medicine, University Hospital of North Durham, Durham, UK; 3 Department of Emergency Medicine, Peterborough City Hospital, Peterborough, PE3 9GZ UK

**Keywords:** Emergency service, hospital, Emergency department, Length of stay, Inpatients, Patient admission

## Abstract

**Background:**

Pressure on emergency departments (EDs) has increased, particularly since the COVID-19 pandemic. There has been limited research into the impact of prolonged emergency department length of stay on inpatient length of stay. This study aimed to evaluate whether a longer stay in the ED increases inpatient length of stay and to quantify any association.

**Methods:**

This was a single-centre, observational, retrospective cohort study of all ED patient episodes that led to inpatient admission from January 2022 to December 2022, at a tertiary hospital in the UK. Separate bivariate analyses were performed for adult and paediatric patients. The effect of multiple confounders for the adult population was evaluated with a logistic regression model. The outcome variable used was an inpatient stay of 7 days, as this is a recognised metric by National Health Service (NHS) England.

**Results:**

From January 2022 to December 2022 there 31 131 admissions from the ED. 31 020 patient attendances were included, representing 23 286 individual patients. For an ED length of stay of less than 12 h, the inpatient length of stay median was 3 days (mean 7.87 days). This rose to 6 days for patients with an ED length of stay of 12 h or greater (mean 9.94 days). Even after adjusting for cofounding variables in adults, an ED length of stay of 12 h of more significantly increases the likelihood of a prolonged inpatient stay (OR 1.30, 95% Confidence Interval (CI) 1.23–1.37), and this effect increased for an ED length of stay of24 h or more (OR 1.50, 95% CI 1.38–1.63). In children, no statistically significant association was observed between ED length of stay and inpatient length of stay for a 12-hour threshold (Pearson Chi² test (9) = 9.4, *p* = 0.405).

**Conclusions:**

This study confirms that there is a significant association between a prolonged ED stay and an increased inpatient stay for patients remaining in the ED over 12 and 24 h.

## Background

The emergency department (ED) is a core part of the UK healthcare system and delivers round the clock emergency care to all patients. This finite resource often becomes the focus of any strain placed on the wider healthcare system. Ageing populations and increased medical complexity have contributed to a rising mismatch between demand and resources [1]. As a result, countries such as the UK are seeing increasing ED length of stay (EDLOS), increased bed occupancy and reduced patient flow [2].

In 2004, the National Health Service England introduced the four-hour access standard for admitting, transferring or discharging ED patients, and in 2010 an operational standard was set at 95% [3]. In 2023, ED performance hit a record low, with only 55% of patients being admitted, transferred or discharged within 4 h from type 1 emergency departments, and the median EDLOS for patients admitted in December 2022 was 7 h 39 min [4]. In the same month, an intermediary four-hour standard of 76% was set to be achieved by March 2024 [5]. The NHS in England has subsequently adopted a 12-hour stay from time of arrival as a recognised metric and key performance indicator. This is reported nationally and monthly, with regulatory penalties for poor performance.

Worldwide studies have already shown the negative associations of long ED stays on 30-day all-cause mortality [6], patient safety events [7], and even delirium [8]. A systematic review from January 2000 to January 2020 found 11 studies looking at EDLOS and inpatient length of stay (IPLOS), of which three focused on patient subsets (sepsis, stroke and head injury) [9]. Seven of the remaining eight papers found a significant positive correlation, however all papers were outside the UK and only four included analyses of confounding factors. From a UK perspective, Paling et al. analysed observational data from all major EDs in England over 90 days in 2016–2017 and found that higher bed occupancy and higher numbers of long-stay admitted patients were independently associated with longer ED waits after adjustment for potential confounders [10].

The pressure on the Urgent and Emergency Care system within the NHS has continued to increase, particularly since the COVID-19 pandemic [2]. There has been limited research into the impact of prolonged EDLOS on IPLOS in the UK. Quantifying this association would inform policy development and clarify whether efforts to reduce EDLOS could lead to operational improvements. Therefore, this study aimed to examine the hypothesis that EDLOS increases IPLOS and to quantify any association, especially after adjustment for multiple confounding variables. It is clinically plausible that a long ED stay with reduced nursing ratios and disrupted medications would increase delirium and fatigue, therefore delaying recovery and increasing length of stay. Work from Taiwan has shown that a 24–48-hour EDLOS was associated with an extra 2.3 days of inpatient stay [11]. A small Canadian study found that increased EDLOS was associated with increased IPLOS, but this was incompletely adjusted for important confounders [12].

A single-centre approach was used as the study centre has had a fully electronic patient record since 2015 and has high quality and little missing data. This allows for more extensive adjustment for confounding variables than would be possible with national datasets. This approach provides a more precise estimation of the association between EDLOS and IPLOS, offering insights that complement and inform broader multi-centre studies.

## Methods

### Study design and setting

This was a single-centre, observational, retrospective cohort study of all patients who arrived at the emergency department and were admitted to an inpatient bed from 1st January 2022 to 31st December 2022 (inclusive) at Addenbrooke’s Hospital, Cambridge, UK. Addenbrooke’s Hospital provides emergency, surgical, and medical care for the local population, and is the major trauma centre for the East of England. A 12-month sample was selected in order to mitigate seasonal fluctuations in attendances and presentations. In 2022, 60.1% of patients left or were admitted from the ED within four hours. All ambulance arrivals were registered as ED patients, regardless of whether the patient could be brought into the department.

### Participants

All patients who attended the ED and were subsequently admitted to Addenbrooke’s Hospital were included in this study, including children. For patients with multiple attendances, each individual episode that resulted in admission was included as independent events. Patients with a 0-minute EDLOS were excluded as they were deemed to represent external pathways and were therefore not representative of true ED exposure. Similarly, patients with an EDLOS over 72 h were excluded, as manual review indicated these cases represented patients attending non-ED pathways, and one patient with manual interference of the arrival time, introducing uncertainty in the recorded timeline. The total number of patients excluded (0-minute and > 72 h patients) represented only 0.36% of the total dataset.

### Data sources and variables

The NHS Health Research Authority Decision Tool was used to assess that ethical approval was not required. The study was registered as a service evaluation with Cambridge University Hospitals NHS Foundation Trust.

Data was gathered from the Trust’s Electronic Patient Record (EPR), which logs all the patient contacts during their stay in the hospital. This information is then securely stored in a centralised database. ED and admission datasets were deterministically linked at an individual patient level, ensuring that EDLOS and IPLOS always refer to the same patient episode. This also enabled inclusion of only those patients admitted through the ED.

The EDLOS and IPLOS from the merged dataset were manually crosschecked using the time of arrival, departure from ED and discharge time. Routinely collected data on potential confounding variables was also reported, including age, gender, arrival from care home, arrival by ambulance, weekend arrival, initial National Early Warning Score (NEWS) 2 score (in adults only), need for isolation and need for critical care admission.

### Statistical methods

There was no sample size calculation calculated prior to analysis. A simple descriptive analysis of the data has been presented. Chi squared tests were conducted for categorical data and a Spearman’s rank correlation for non-parametric continuous data.

The effect of multiple confounders has been evaluated with a logistic regression model by transforming most potential confounders, e.g. ‘arrival from care home’ or ‘need for critical care admission’, into binary variables. Age was transformed into 10-year bands, starting at 16 years of age, and finishing with 85 years and over. These bands were treated as multiple dichotomous variables. The outcome variable used was an inpatient stay of 7 days, as this is a recognised metric by NHS England. The regression model was used to adjust for the effect of confounding variables on 12-hour and 24-hour stays.

Separate bivariate analyses were performed for adult and paediatric patients (under the age of 16 years old), reflecting the expectation that these populations would be managed through distinct departments and clinical pathways. The regression analysis depended on the NEWS2 values, which are not applicable to paediatric patients. Therefore, patients under 16 years old were excluded from the regression analysis. All analyses were conducted using Stata, version 18 [13].

## Results

During the study period there were 136 464 attendances in the ED, resulting in 31 131 admissions. In total, 111 attendances (0.36%) were excluded due to an EDLOS of 0-minutes (68) and 72 h or greater (43) EDLOS. This left 31 020 patient attendances for inclusion, representing 23 286 individual patients. Therefore approximately 25% of admissions were repeat admissions. Patients under 16 constituted 18.7% of the dataset (5823 attendances). A bivariate analysis of EDLOS in minutes and IPLOS in days and demonstrated a moderate correlation (Spearman’s rho 0.196, *p* < 0.001).

Table 1 shows the demographic characteristics of this cohort. The median age of patients admitted from the ED was 69 years. 50% arrived by ambulance, and 7.4% originated from a care home. The most frequent time of arrival was between 12:00 and 17:59, and approximately 25% of admissions occurred at the weekend. Attendances resulting in admission were relatively evenly distributed across the four quarters of the year (range 24.64% to 25.44%).

**Table 1:** Demographic, temporal and clinical characteristics of patients admitted from the ED

Patient characteristics	Number	Percentage (%)
Age (years)		
0–15	2636	8.50
16–24	1651	5.32
25–34	2006	6.47
35–44	2162	6.97
45–54	2652	8.55
55–64	3269	10.54
65–74	4266	13.75
75–84	6555	21.13
> 85	5823	18.77
Male	15,015	48.40
Female	16,003	51.59
Arrival by Ambulance	15,468	49.86
Arrival from Care Home	2293	7.39
Isolation requirement	5140	16.57
Critical Care Stay	1465	4.72
Time of arrival		
00:00–05:59	3428	11.05
06:00–11:59	7355	23.71
12:00–17:59	11,868	38.26
1800–2359	8369	26.98
Day of Arrival		
Weekend	7867	25.36
Weekday	23,153	74.64
Month of Arrival		
January - March	7642	24.64
April - June	7794	25.13
July - September	7691	24.79
October - December	7893	25.44
First NEWS2 Score (Adults)		
0	7395	26.05
1	6749	23.78
2	4213	14.84
3	3096	10.91
4	1981	6.98
5	1433	5.05
6	1026	3.61
7	719	2.53
8	555	1.96
9	420	1.48
No score	797	2.81

The majority of patients were admitted under medical specialities (60.34%). A need for isolation was recorded in 16.57% of admission from ED and 4.72% required admission to critical care at some stage of their inpatient stay. The mean time that a patient spent in the ED was approximately 9 h with only 19.28% of patients meeting the 4-hour standard set by the NHS. The mean length of stay as an inpatient was 4 days.

Further analysis of the demographic, clinical, and temporal characteristics for patients is provided in Table 2, comparing those with an EDLOS less than 12 h, and an EDLOS of 12 h or longer. Patients with an EDLOS of less than 12 h had a median IPLOS of 3 days (mean 7.87 days), rising to 6 days for patients with an EDLOS of 12 h or longer (mean 9.94 days).

**Table 2:** Comparison of characteristics for adults ED length of stay less than 12 h compared to 12 h or more

	ED length of stay < 12 h		ED length of stay > = 12 h	
	Number	Percentage (%)	Number	Percentage (%)
Number of patients	19,462	62.74	11,558	37.26
Male	9700	49.84	5315	45.99
Female	9761	50.15	6242	54.01
Arrival by ambulance	8544	43.90	6924	59.91
Arrival from care home	1134	5.83	1159	10.03
Isolation requirement	3506	18.01	1634	14.14
Critical Care Stay	1199	6.16	266	2.30
Arrival:				
00:00–05:59	1973	10.14	1455	12.59
06:00–11:59	5055	25.97	2300	19.90
12:00–17:59	7793	40.04	4075	35.26
1800–2359	4641	23.85	3728	32.25
Weekend	5411	27.80	2456	21.25
Weekday	14,051	72.20	9102	78.75
January - March	5267	27.06	2375	20.55
April - June	5081	26.11	2713	23.47
July - September	4554	23.40	3137	27.14
October - December	4560	23.43	3333	28.84
	**Mean**	**Median**	**Mean**	**Median**
Age	57.05	63	68.46	75
First NEWS Score	2.07	1	2.23	2
IP LOS	7.87	3	9.94	6

A bivariate analysis of EDLOS and IPLOS in the total study population (Table 3) demonstrated a significant association between longer EDLOS and increased IPLOS (Pearson Chi² test (9) > 1000, *p* < 0.0001). This pattern held true when EDLOS was dichotomised at both 12-hour and 24-hour thresholds.

**Table 3:** Bivariate analysis of ED length of stay and inpatient length of stay in the total population (adults and paediatric)

**Inpatient Length of Stay (days)**	**ED Length of Stay** **< 12 h**	**ED Length of Stay** **>=12 h**	**Total** (***n***)
0	2,444	495	2,939
1	3,628	1,378	5,006
2	2,366	1,192	3,558
3	1,601	1,065	2,666
4	1,265	855	2,120
5	1,042	752	1,794
6	857	649	1,506
7–13	3,188	2,637	5,825
14–20	1,324	1,117	2,441
21 to maximum	1,747	1,418	3,165
Total	19,462	11,558	31,020
Pearson Chi2 test (9) > 1,000 *p* > 0.0001			
**Inpatient Length of Stay (days)**	**ED Length of Stay** **< 24 h**	**ED Length of Stay** **>= 24 h**	**Total** **(n)**
0	2,925	14	2,939
1	4,808	198	5,006
2	3,266	292	3,558
3	2,390	276	2,666
4	1,878	242	2,120
5	1,589	205	1,794
6	1,317	189	1,506
7–13	5,066	759	5,825
14–20	2,135	306	2,441
21 to maximum	2,753	412	3,165
Total	28,127	2,893	31,020
Pearson Chi2 test (9) = 665 *p* > 0.0001			

**Fig. 1:**
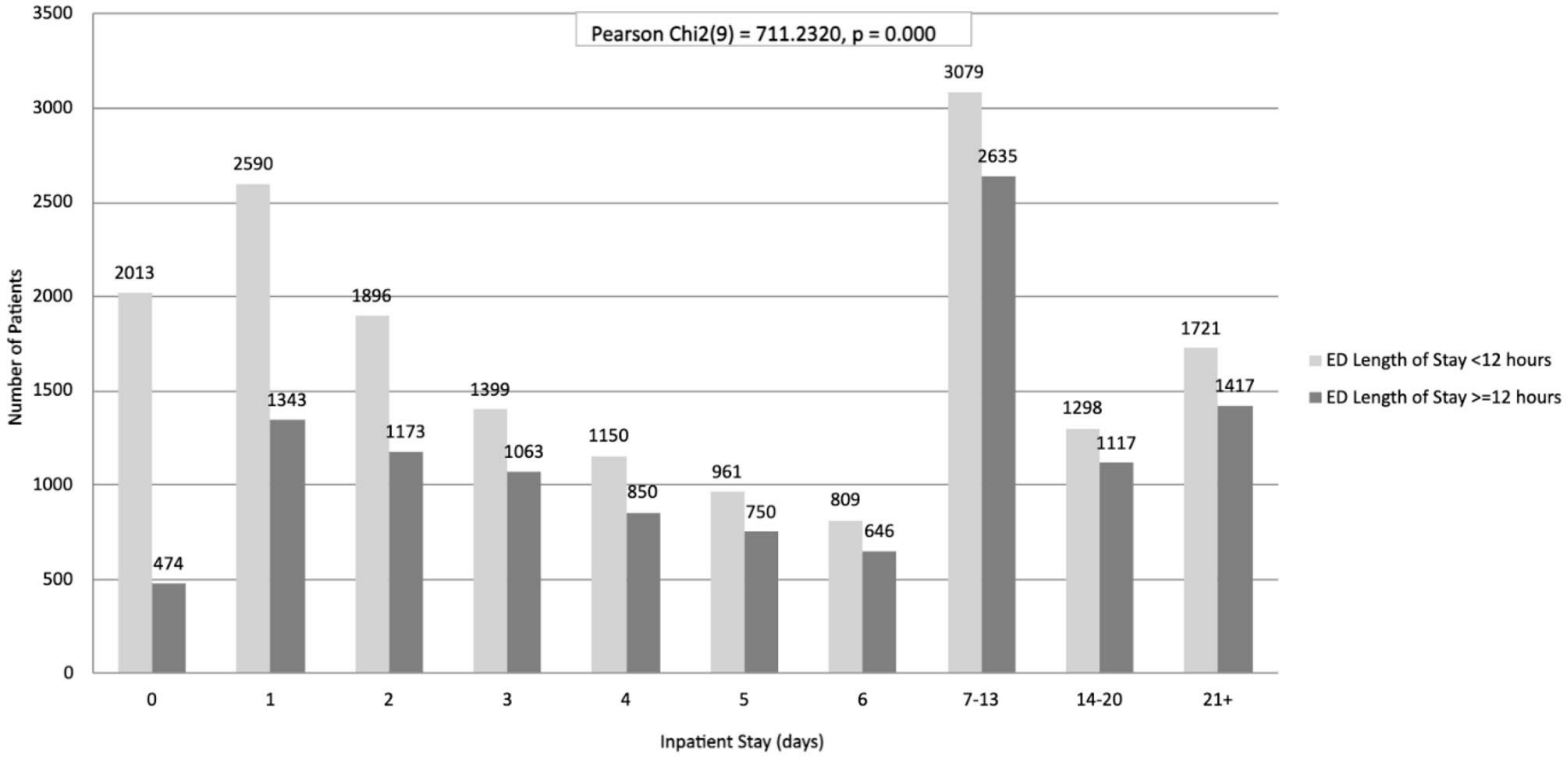
Adult population bivariate analysis of ED length of stay and inpatient length of stay using a 12-hour threshold

**Fig. 2:**
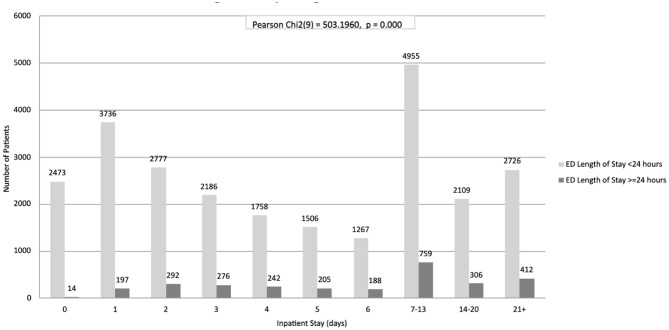
Adult population bivariate analysis of ED length of stay and inpatient length of stay using a 24-hour threshold

In the adult subgroup (Table 4), this association remained statistically significant, with longer EDLOS significantly associated with prolonged inpatient stay at both the 12-hour threshold (Pearson Chi² test (9) = 711.2, *p* < 0.0001; Fig. 1) and the 24-hour threshold (Pearson Chi² test (9) = 503.2, *p* < 0.0001; Fig. 2).

**Table 4:** Adult population bivariate analysis of ED length of stay and inpatient length of stay

**Inpatient Length of Stay (days)**	**ED Length of Stay** **< 12 h**	**ED Length of Stay** **>=12 h**	**Total** (***n***)
0	2,013	474	2,487
1	2,590	1,343	3,933
2	1,896	1,173	3,069
3	1,399	1,063	2,462
4	1,150	850	2,000
5	961	750	1,711
6	809	646	1,455
7–13	3,079	2,635	5,714
14–20	1,298	1,117	2,415
21 to maximum	1,721	1,417	3,138
Total	16,916	11,468	28,384
Pearson Chi2 test (9) = 711.2, *p* < 0.0001			
**Inpatient Length of Stay (days)**	**ED Length of Stay** **< 24 h**	**ED Length of Stay** **>= 24 h**	**Total** **(n)**
0	2,473	14	2,487
1	3,736	197	3,933
2	2,777	292	3,069
3	2,186	276	2,462
4	1,758	242	2,000
5	1,506	205	1,711
6	1,267	188	1,455
7–13	4,955	759	5,714
14–20	2,109	306	2,415
21 to maximum	2,726	412	3,138
Total	25,493	2,891	28,384
Pearson Chi2 test (9) = 503.2, *p* < 0.0001			

**Fig. 3:**
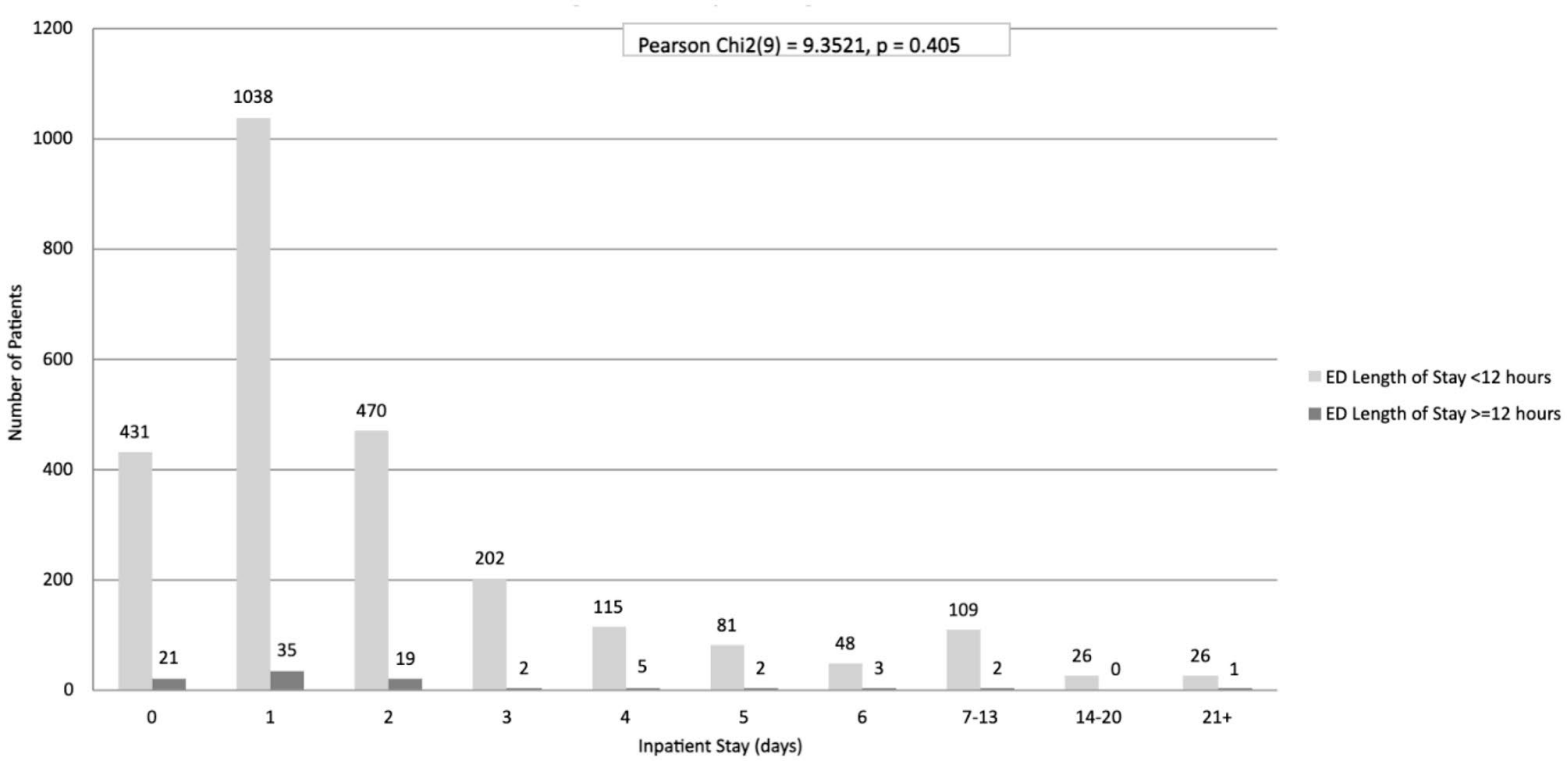
Paediatric population bivariate analysis of ED length of stay and inpatient length of stay using a 12-hour threshold

In the paediatric subgroup (Table 5), analysis at the 12-hour threshold showed a weak trend indicating that a longer EDLOS may be associated with a shorter IPLOS. However, this potential inverse relationship was based on a small number of cases, and did not reach statistical significance (Pearson Chi²(9) = 9.4, *p* = 0.405; Fig. 3). Furthermore, analysis at the ≥ 24-hour threshold was not feasible due to the extremely limited number of patients in that category (*n* = 2).

**Table 5:** Paediatric Population bivariate analysis of ED length of stay and inpatient length of stay

**Inpatient Length of Stay (days)**	**ED Length of Stay** **< 12 h**	**ED Length of Stay** **>=12 h**	**Total** (***n***)
0	431	21	452
1	1,038	35	1,073
2	470	19	489
3	202	2	204
4	115	5	120
5	81	2	83
6	48	3	51
7–13	109	2	111
14–20	26	0	26
21 to maximum	26	1	27
Total	2546	90	2,636
Pearson Chi2 test (9) = 9.4, *p* = 0.405			
**Inpatient Length of Stay (days)**	**ED Length of Stay** **< 24 h**	**ED Length of Stay** **>= 24 h**	**Total** **(n)**
0	452	0	452
1	1,072	1	1,073
2	489	0	489
3	204	0	204
4	120	0	120
5	83	0	83
6	50	1	51
7–13	111	0	111
14–20	26	0	26
21 to maximum	27	0	27
Total	2,634	2	2,636
Pearson Chi2 test not performed as assumptions not met			

**Fig. 4:**
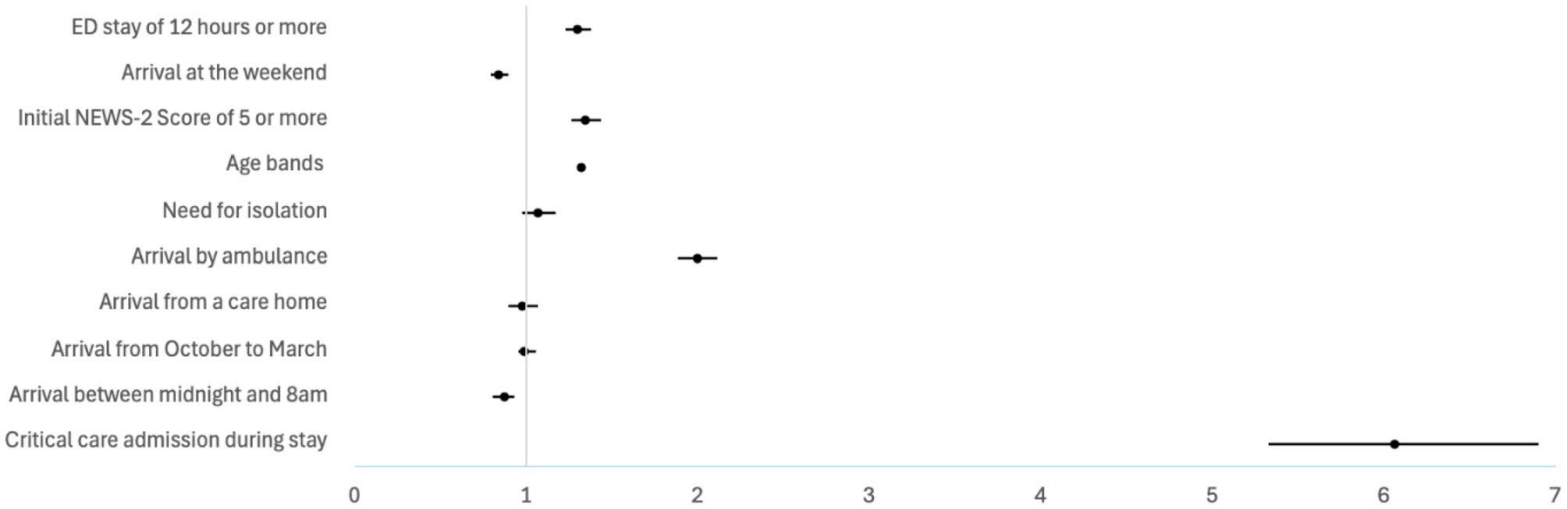
Adjusted odds ratio for predictors of inpatient length of stay > 7 days in adults using ED stay threshold of 12 h. Forest plot showing adjusted odds ratios (95% Confidence Intervals) for predictors of inpatient length of stay > 7 days in adults, based on multivariable logistic regression models using ED stay thresholds of 12 h

**Fig. 5:**
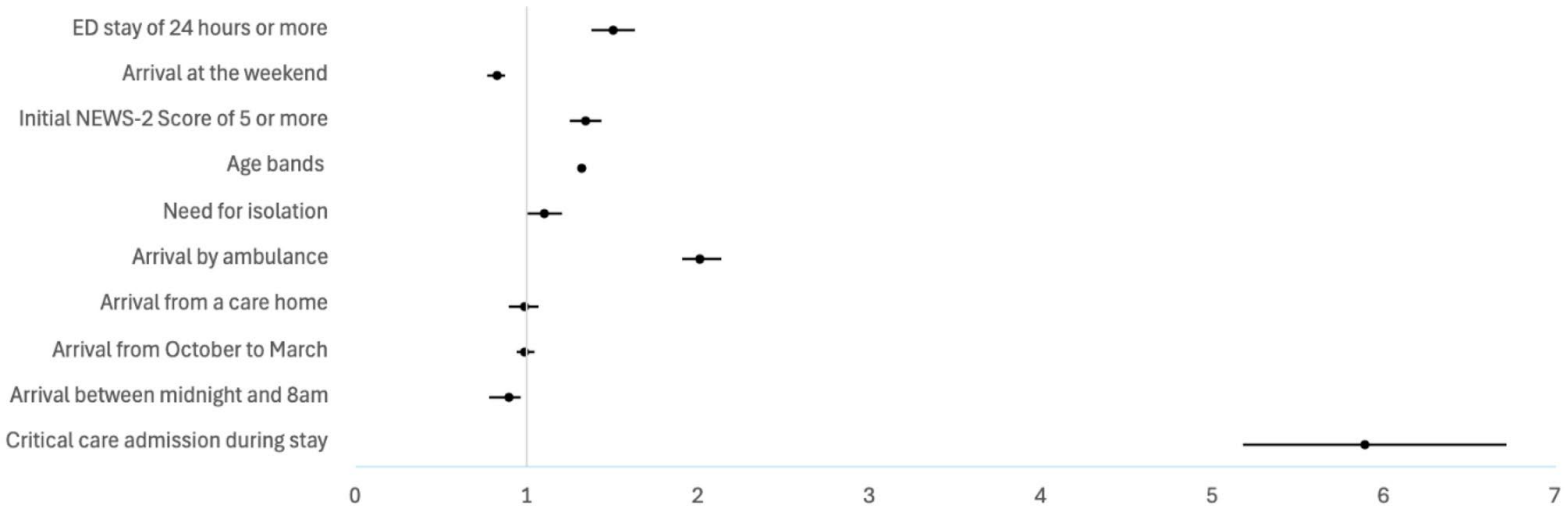
Adjusted odds ratio for predictors of inpatient length of stay > 7 days in adults using ED stay threshold of 24 h. Forest plot showing adjusted odds ratios (95% Confidence Intervals) for predictors of inpatient length of stay > 7 days in adults, based on multivariable logistic regression models using ED stay thresholds of 24 h

Table 6 shows a multivariate logistic regression analysis of predictors associated with the risk of an IPLOS greater than 7 days in adults. An EDLOS of 12 h of more significantly increases the likelihood of a prolonged inpatient stay (OR 1.30, 95% CI 1.23–1.37; Fig. 4), and this effect increases for an EDLOS of 24 h or more (OR 1.50, 95% CI 1.38–1.63; Fig. 5). Critical care admission and arrival by ambulance were the strongest predictors of prolonged inpatient stays, followed by a high NEWS-2 score and increasing age bands. Weekend and night arrivals appeared to be protective factors, reducing the likelihood of extended hospitalisation.

**Table 6:** Multivariate logistic regression analysis: predictors of inpatient length of stay of more than 7 days for adults

	Odds Ratio	Standard Error	z	*p* value	95% Confidence Intervals
**ED stay of 12 h or more**	1.30	0.03	9.65	0.00	1.23–1.37
Arrival at the weekend	0.84	0.03	-5.89	0.00	0.79–0.89
Initial NEWS-2 Score of 5 or more	1.34	0.04	8.98	0.00	1.26–1.43
Age bands	1.32	0.01	41.25	0.00	1.30–1.34
Need for isolation	1.07	0.05	1.4	0.16	0.97–1.17
Arrival by ambulance	1.99	0.06	23.85	0.00	1.88–2.11
Arrival from a care home	0.97	0.05	-0.56	0.58	0.89–1.07
Arrival from October to March	0.99	0.03	-0.19	0.85	0.95–1.05
Arrival between midnight and 8am	0.87	0.03	-3.78	0.00	0.80–0.93
Critical care admission during stay	6.06	0.40	27.02	0.00	5.32–6.90
**ED stay of 24 h or more**	1.50	0.06	9.67	0.00	1.38–1.63
Arrival at the weekend	0.82	0.02	-6.53	0.00	0.77–0.87
Initial NEWS-2 Score of 5 or more	1.34	0.04	8.90	0.00	1.25–1.43
Age bands	1.32	0.01	41.98	0.00	1.31–1.34
Need for isolation	1.10	0.05	1.97	0.05	1.00-1.20
Arrival by ambulance	2.01	0.06	24.19	0.00	1.9–2.13
Arrival from a care home	0.98	0.05	-0.48	0.63	0.89–1.07
Arrival from October to March	0.99	0.03	-0.41	0.68	0.94–1.04
Arrival between midnight and 8am	0.89	0.05	-2.20	0.03	0.78–0.96
Critical care admission during stay	5.89	0.05	26.61	0.01	5.17–6.71

## Discussion

### Key results

This study has evaluated the association between ED length of stay and inpatient length of stay. The results show a positive association for adults that persists even after extensive adjustment for multiple confounding variables. Adult patients with an ED stay of 12 h or more have a 1.3 Odds ratio of being admitted for 7 days or longer when compared to those with a shorter ED stay. This risk increases to 1.5 for those with an ED stay of 24 h or more. For paediatric patients, a longer EDLOS was weakly associated with a shorter IPLOS. This suggests care may be completed in the ED that would otherwise have constituted a proportion of the inpatient stay.

It has also demonstrated that a 24-hour stay in the ED is associated with a reduction in inpatients stays lasting 0 or 1 days. This is despite the clock for the inpatient admission not starting until the patient arrives on an inpatient ward. This can be explained by patients completing the majority of a short stay admission in the ED, and also by patients requiring a longer inpatient stay after a 24-hour stay in ED.

The strongest predictors of an IPLOS longer than 7 days in adults were critical care admission, arrival by ambulance, an initial NEWS 2 score of 5 or higher, and increasing age bands. Arrival from a care home showed a small but statistically significant reduction in IPLOS, suggesting that discharge from an inpatient bed may be easier if a patient has a care home to go to.

Temporal analysis showed arrival overnight or arrival from October to March had no statistically significant impact. Arrival between midnight and 8am was shown to be a protective factor, perhaps due to earlier inclusion in a consultant-led ward round. Arrival at the weekend in fact reduced IPLOS. The reason for this is unclear but hypotheses would include differences in casemix, senior staffing or thresholds for admission.

### Limitations

This study has some important limitations. It was conducted in a single tertiary centre and the external validity of these findings is uncertain. While this setting allowed for detailed patient-level analysis and minimised missing data through a fully paperless EPR system, the results may not be generalisable to healthcare environments with different patient demographics, resource availability, or organisational structures.

The limitations of routine data are well described, but the electronic patient record used allowed for high quality, automated data capture. The total number of excluded patients made up a negligible proportion of the data set and is unlikely to introduce meaningful bias or influence the study findings. It was beyond the scope and objectives of this study to adjust for staffing skill mix and numbers.

There are some interactions between the confounding variables; however, the variables appear to measure substantially different domains, and the risk of over-adjustment is deemed limited. For example, a potential limitation in this analysis lies in the adjustment both initial NEWS score and critical care admission. These variables are not truly independent as NEWS score is designed to predict clinical deterioration. This leaves potential for over-adjustment which could underestimate the impact of prolonged ED stay on IPLOS. There may be an effect from other confounding variables that are not routinely collected and therefore not included in our model.

However, in this patient cohort, only 27.13% of patients admitted to critical care had high initial NEWS scores (7 or higher). Nearly half (49.53%) of patients requiring critical care had low initial NEWS scores, suggesting that factors other than early physiological deterioration contributed to the critical care admission. This distribution suggests that while there is some overlap, NEWS score and critical care admission capture distinct aspects of patient acuity and clinical course, justifying their inclusion as separate adjustment variables in the analysis. The data does not allow capture of whether these patients were admitted to critical care directly from the emergency department, following an inpatient stay, or following a procedure.

Patients with multiple attendances were included in this study, which increased the sample size and statistical significance while also capturing the true spectrum of ED attendances. However, this may lead to overrepresentation of high intensity users, whose complex health needs could independently influence both EDLOS and IPLOS. This introduces a potential bias when visits are not independent of each other. Furthermore, patients with repeat attendances which convert to admission may have more severe underlying health conditions.

ED stays of 0 min and over 72 h were removed to improve data reliability. The authors are confident that the cases excluded represented inconsistent data but acknowledge that anomalies may extend beyond the cut offs set given the complexity of this dataset. The amount of excluded data was small (0.36%) and unlikely to have biased results.

### Implications

These findings provide further evidence of how ED pressures are likely to have a domino effect on patient outcomes and the wider healthcare system. From an operational perspective, longer inpatient stays would be expected to negatively impact bed occupancy, patient flow, and healthcare costs. From a clinical perspective, prolonged hospitalisation may increase the risk of hospital-acquired infections, contributes to functional decline, and impact patient satisfaction, ultimately affecting long-term morbidity and overall care quality. Targeted interventions to prioritise ED patient flow and improve patient admission pathways may enhance patient care, reduce inpatient length of stay, improve resource utilisation and reduce healthcare expenditure [14–16].

While this study provides valuable insights from a single-centre, future research on the association between EDLOS and IPLOS could be conducted at a multi-centre or national level in order to test the external validity of these findings. Adjustment for ED staffing and crowding may also give greater insights into causation. Further research into the effect of EDLOS on mortality in a UK setting is needed to further determine the impact of physiological variables.

## Conclusion

This study highlights a significant association between a prolonged ED length of stay and an increased inpatient length of stay for patients remaining in the ED over 12 h. The association persists after adjustment for key confounding variables. Addressing pressure in the ED and enhancing patient flow may have a positive impact on patient care and resource management.

## Data Availability

The datasets generated and/or analysed during the current study are not publicly available due to patient confidentiality and restrictions imposed by the data governance policies of Cambridge University Hospitals NHS Foundation Trust, but are available from the corresponding author on reasonable request and subject to appropriate approvals.

## Abbreviations

ED: Emergency Department
EDLOS: Emergency Department Length of Stay
IPLOS: Inpatient Length of Stay
NHS: National Health Service
NEWS2: National Early Warning Score 2
EPR: Electronic Patient Record
CI: Confidence Interval
OR: Odds Ratio
UK: United Kingdom
COVID-19: Coronavirus Disease 2019
